# Multiple environmental controls explain global patterns in soil animal communities

**DOI:** 10.1007/s00442-020-04640-w

**Published:** 2020-04-07

**Authors:** Alice S. A. Johnston, Richard M. Sibly

**Affiliations:** School of Biological Sciences, University of Reading, Reading, RG6 6AH UK

**Keywords:** Community composition, Mass–abundance, Size spectra, Soil animals, Soil organic carbon, Soil pH, Temperature

## Abstract

Soil animals play important roles in ecosystem functioning and stability, but the environmental controls on their communities are not fully understood. In this study, we compiled a dataset of soil animal communities for which the abundance and body mass of multiple soil animal groups were recorded. The mass–abundance scaling relationships were then used to investigate multiple environmental controls on soil animal community composition. The data reveal latitudinal shifts from high abundances of small soil animals at high latitudes to greater relative abundances of large soil animals at low latitudes. A hierarchical linear mixed effects model was applied to reveal the environmental variables shaping these latitudinal trends. The final hierarchical model identified mean annual temperature, soil pH and soil organic carbon content as key environmental controls explaining global mass–abundance scaling relationships in soil animal communities (*R*^2^_c_ = 0.828, *N*_group_ = 117). Such relationships between soil biota with climate and edaphic conditions have been previously identified for soil microbial, but not soil animal, communities at a global scale. More comprehensive global soil community datasets are needed to better understand the generality of these relationships over a broader range of global ecosystems and soil animal groups.

**Electronic supplementary material:**

The online version of this article (10.1007/s00442-020-04640-w) contains supplementary material, which is available to authorized users.

## Introduction

Soil animals play important roles in ecosystem functions such as carbon and nutrient cycling, water regulation and primary production. The composition of soil animal communities thus strongly influences ecosystem multifunctionality (Wagg et al. 2014). For instance, soil animal communities alter microbial activity, litter decomposition, nutrient mineralization and soil respiration rates, and plant community composition (Bradford et al. 2002; De Deyn et al. 2003; Eisenhauer et al. 2012a, b; Johnston and Sibly 2018). Soil animal groups, of different body size ranges, also display divergent responses to global environmental changes (Blankinship et al. 2011; Eisenhauer et al. 2012a, b). Shifts in soil animal community composition could thus have dramatic consequences for terrestrial ecosystem functioning and stability in the future (Sjursen et al. 2005; Suttle et al. 2007; Briones et al. 2009; Eisenhauer et al. 2014; Handa et al. 2014). Yet, little is known about the environmental controls that shape entire soil animal communities at a global scale.

Petersen and Luxton (1982), in the most comprehensive synthesis of soil animal abundances and biomasses to date, revealed non-linear shifts in soil animal community biomass with latitude. Total soil animal biomass declined from temperate ecosystems (forests and grasslands) towards both arctic and tropical ecosystems and were accompanied by shifts in soil animal community composition. For instance, the biomass of smaller soil animal groups (Nematoda, Collembola, Enchytraeidae and Acari) increased in arctic and decreased in tropical, relative to temperate, ecosystems. In a more recent synthesis, Fierer et al. (2009) compiled biomass measurements for five soil animal groups (Nematoda, Acari, Collembola, Enchytraeidae and Oligochaeta) from different studies and found similar variations in soil animal community composition across seven biomes. The underlying environmental controls shaping latitudinal shifts in soil animal communities at a global scale, however, have not been identified. Our study aims to address this knowledge gap by synthesising data for entire soil animal communities and the environmental controls that influence their composition across globally distributed sites.

Climatic constraints are classically considered the key drivers of soil animal distribution and abundance (Wardle et al. 2004). For instance, a global litter decomposition study found the influence of soil animals to be climate dependent (Wall et al. 2008). Recent global syntheses of soil animal groups further identify climate as an important driver of nematode (Van Den Hoogen et al. 2019) and earthworm (Johnston 2019; Phillips et al. 2019) community metrics. Yet, inconsistent observations from soil warming experiments suggest that climate effects on soil animals are subsidiary to resource effects at the community and site level (Ernakovich et al. 2014; Thakur et al. 2014; Dorrepaal et al. 2016; Robinson et al. 2018). Resource availability and diversity influence soil animals by altering the amount of energy and nutrients available for consumption (Mueller et al. 2016; Van der Wal et al. 2009), and opportunities for niche differentiation between soil animal groups (Wardle 2006). Soil animals, however, consume diverse resources, including plant roots, leaf litter, microorganisms and one another. Nevertheless, strong coupling relationships exist between plant productivity and soil animal communities (Yang et al. 2018), suggesting shared environmental controls on plant and soil animal communities.

Nutrients play a central role in plant allocation strategies. When nitrogen (N) and/or phosphorous (P) are limiting, photosynthate C is allocated preferentially belowground to root growth to compete for limited nutrients, and otherwise allocated aboveground to shoot and leaf growth to compete for limiting light (Gill and Finzi 2016). Plant allocation above- or belowground has consequences for soil communities, by directing the availability of food resources to different soil animal groups. A regional study of the body-size spectra of soil bacteria, fungi and micro- (Nematoda) and meso-fauna (Acari, Collembola, Enchytraeidae) in 22 Dutch grasslands, for instance, found greater N and P availability to support higher abundances of the larger soil animals sampled in the study (Mulder and Elser 2009). These observations suggest that greater aboveground plant production, related to higher soil nutrient contents, will support the presence, and greater abundances of larger soil animal groups (macrofauna, e.g. Chilopoda, Clitellata) at a global scale. Conversely, we would expect nutrient limited soils, where plant production is directed mainly belowground, to harbour greater abundances of smaller soil animal groups (e.g., micro- and meso-fauna).

Global patterns in soil microbial communities have received much more attention than soil animals, and evidence suggests that the relative abundance and biodiversity of bacteria and fungi groups are strongly influenced by both climate and soil nutrients (Treseder 2008; Serna‐Chavez et al. 2013; Koyama et al. 2014; Tedersoo et al. 2014; Delgado-Baquerizo et al. 2017). Given the global relationships between climate and plant nutrient stoichiometry with litter decomposition rates (Parton et al. 2007) and the influence of soil animals on decomposition (García‐Palacios et al. 2013), we hypothesise that these environmental controls (climate and nutrients) extend to soil animal communities. To test this hypothesis, we conducted a comparative analysis of global soil animal communities. First, we compiled a dataset of soil animal communities for which abundance and biomass were recorded for multiple soil animal groups across globally distributed sites. We then characterised soil community composition by mass–abundance scaling relationships to investigate global shifts in soil animal body-size spectra. Finally, a hierarchical linear mixed effect model was used to test the importance of multiple environmental controls on global soil animal communities.

## Materials and methods

### Synthesis of soil animal community data

Soil animal community studies were synthesised from the literature using Web of Science (www.webofknowledge.com). To be included in the dataset, studies had to report field-collected abundances and biomass or body masses for at least four soil animal groups (to be representative of the soil animal community) in un-manipulated field conditions. Search terms included soil or belowground, animal, fauna, biota, decomposer, invertebrate or detrivore, community, and excluded the terms laboratory, microcosm or mesocosm in the title. Additional search terms for the topic included: population or community; abundance, density, number or biomass; and field, forest, grassland, or natural. Field studies were further excluded if they did not report site information such as latitude, longitude or site location, or the year for the extraction of climatic data. Most studies evaluated, in total 54, did not include both abundance and biomass endpoints or only studied a few soil animal groups.

Twelve studies met the search criteria, reporting thirteen soil animal communities in ecosystems ranging from arctic tundra to tropical rainforest (Table 1). Soil animal groups include Nematoda, Collembola, Enchytraeidae, Acari, Diptera, Hymenoptera, Coleoptera, Isopoda, Chilopoda, Araneae, Blattodea, Diplopoda, Gastropoda, Clitellata and Other Insecta groups (Table 2). Detail at the species level was avoided so that macroecological patterns in soil animal community composition could be evaluated. The raw soil community dataset (*N*_raw_ = 1503), which in some cases reported seasonal or annual soil community dynamics (abundance and biomass), were summed across families and/or species of the same soil animal group and/or averaged across study year/s. The summarised dataset provides 117 data points, relating the abundance and body mass of each sampled soil animal group per study.

**Table 1:** Summary of the soil animal community studies included in the dataset

Study	Location	Vegetation	Soil type
Anderson et al. (1983)	Sarawak, Malaysia 4.37° N, 113.97° E	Dipterocarp rainforest	Well-drained organic soil over limestone
Axelsson et al. (1984)	Andersby, Sweden 60.15° N, 17.83° E	Oak–hazel woodland	Glacial boulder till
Byzova, Uvarov and Petrova (1995)	Spitsbergen, Svalbard 77.00° N, 15.55° E	Arctic tundra (*Salix polaris*)	Gelic Gleysol
Coulson and Whittaker (1978)	Cumbria, England 54.65° N, 2.45° W	^a^Limestone (*Festuca–Agrostis*) grassland	Red-brown limestone soil
		^b^Acid (*Juncus squarrosus*) moorland	Redistributed peat
Dial et al. (2006)	Sabah, Malaysia 4.00° N, 117.00° E	Dipterocarp rainforest	Orthic acrisol
Hoste-Danyłow et al. (2013)	Kampinos National Park, Poland 52.35° N, 20.55° E	Deciduous (*Quercus robur*) forest	Glaciofluvial and fluvial sands
Huhta and Koskenniemi (1975)	Hameenlinna, Finland 62.67° N, 26.00° E	Myrtillus-type spruce forest	Moraine with podzol
Moulder and Reichle (1972)	Tennessee, USA 35.93° N, 84.31° W	Poplar (*Liriodendron*) forest	Colluvial deposits over limestone
Rosswall, Persson and Lohm (1977)	Uppsala, Sweden 60.00° N, 17.19° E	Abandoned field	Fen peat soil
Persson et al. (1980)	Ivantjarnsheden, Sweden 60.46° N, 16.39° E	Scots pine (*Pinus sylvestris*) forest	Iron podzol
Richardson et al. (2005)	El Verde, Puerto Rico 18.32° N, 65.82° W	Neotropical (*Cyrilla racemiflora*) forest	Clay loam
Xu et al. (2017)	Dongling Mountain, China 40.00° N, 115.00° E	Mixed (*Q. liaotungensis*) forest	Brown soil

**Table 2:** Summary of the mean body mass and abundance (with ± standard errors) for each soil animal group in the study, indicating each groups representation in the summarised (*N*_group_) and raw (*N*_raw_) dataset, and the lower taxonomic groups included (where applicable)

Soil animal group	Body mass (mg dw)	Abundance (ind m^−2^)	*N*_group_	*N*_raw_	Lower taxanomic groups included
Acari	0.0073 ± 0.0015	134,977 ± 72,416	9	230	Gamasina, Oribatida, Mesostigmata
Araneae	1.10 ± 0.61	3032 ± 2858	10	157	Linyphiidae, Lycosidae, Gnaphosidae, Clubionidae
Blattodea	3.60 ± 1.69	253.12 ± 233	4	24	Isoptera
Chilopoda	4.82 ± 2.91	188.10 ± 125	8	32	Geophilomorpha, Lithobiomorpha
Coleoptera	13.95 ± 10.22	289.12 ± 98.19	11	312	Staphylinidae, Scydmaenidae, Carabidae
Collembola	0.0335 ± 0.0234	37,336 ± 9979	12	221	Tullbergiidae, Isotomidae, Entomobryomorpha, Symphypleona, Onychiuridae
Diplopoda	17.99 ± 5.30	52.26 ± 30.50	8	30	Julida, Polydesmida
Diptera	6.16 ± 4.20	1632.33 ± 770.81	8	179	
Enchytraeidae	0.0720 ± 0.0264	48,972 ± 27,351	7	20	
Gastropoda	30.94 ± 10.82	10.33 ± 2.96	5	17	Pulmonata
Hymenoptera	0.6928 ± 0.2059	559.55 ± 324.96	7	46	Formicidae
Isopoda	5.87 ± 4.82	81.98 ± 56.28	5	8	Porcellionidae
Nematoda	0.0001 ± 0.0001	3,369,911 ± 769,473	5	21	Adenophorea, Secernentea
Clitellata	35.53 ± 8.32	220.32 ± 96.02	7	77	Oligochaeta, Lumbricina, Moniligastrida, Hirudinea
Other Insecta	10.47 ± 6.01	241.81 ± 136.76	11	129	Hemiptera, Orthoptera, Staphylinidae, Dermaptera, Diplura, Thysanoptera

Abundances were typically reported as individuals m^−2^, and biomasses as mg or g dry weight m^−2^_._ Two studies (Huhta and Koskenniemi 1975; Anderson et al. 1983) reported biomasses as fresh weight, and here generic conversion ratios for 11 soil animal groups were compiled from the literature. Dry mass contents were taken as 0.27, 0.43, 0.43, and 0.45 for Araneae, Hymenoptera, Coleoptera, and ‘other insect’ groups, respectively (Sage 1982); 0.40, 0.35, 0.18, and 0.18 for Acari, Collembola, Enchytraeidae and Clitellata (Törmälä^1979^); 0.18 for Gastropoda (Lyth 1982) and 0.31 for Chilopoda and Diplopoda (Hadley 1982), and 0.27 for Blattodea (Redford and Dorea 1984).

### Environmental variables

Environmental variables for the thirteen sites sampled in the soil fauna community dataset were compiled, if available, from the respective studies. However, the majority of studies did not report the full scope of environmental variables included in the dataset (latitude, mean annual temperature (MAT), mean annual precipitation (MAP), soil moisture, pH, soil organic carbon, total soil N, total soil P, soil C:N, soil N:P, soil C:P and litter layer). These data gaps were addressed by searching the literature for other measurements made at the same sample sites (for details, see Table S1 in the Supporting Information). Preference was given to data that were reported within 5 years of the study dates, but in some cases, the time gap had to be extended. When soil properties were reported according to soil layer, an average value for all soil layers was calculated. Although data borrowing in this way will introduce error, it was deemed the most appropriate way to fill data gaps in soil properties as global mapping tools such as the ISRIC SoilGrids database do not offer a fine resolution. MAT and MAP, if not reported, were compiled from local NOAA weather stations (www.ncdc.noaa.gov/cdo-web/datatools/findstation) or UK Met Office stations (www.metoffice.gov.uk/public/weather/climate-historic/) using reported latitude, longitude and study year/s.

### Statistical analyses

Scaling relationships between soil animal body masses (*M*, mg dw) and abundances (*A*, ind m^−2^) were used to characterise the body-size spectra of soil animal communities. Mass–abundance relationships were first analysed according to a linear model, without interaction terms or random effects. Then interactions between *M* and climate (arctic, boreal, temperate, tropical), ecosystem type (forest or grassland), study/site (thirteen groups) and latitude (° N) were tested to uncover general patterns in global soil animal communities.

Hierarchical models were then used to test the importance of environmental controls on soil animal community composition across globally distributed sites. In this way, our analysis aimed to uncover the environmental variables structuring soil communities at a global scale. All models were linear mixed effects models, with random effects assigned to the study (*N*_study_ = 13), and the soil animal group sample size which varied both across studies and soil fauna groups (mean sample size: 12.85 ± 2.94, *N*_groups_ = 117). All statistical analyses were performed in R version 3.5.1 (R Core Team 2018), using the lme4 package and lmer function for linear mixed effects models.

The hierarchy of terms tested followed an order similar to that suggested by de Vries et al. (2012), in which ‘controls’ are added before ‘function’. That is, variables that cause variations in multiple soil properties and plant production (e.g., climate) are added first, so that if ‘controls’ explain the variation in ‘functions’ then addition of these variables do not improve model likelihood. Terms were added in the order: climate (MAT and MAP), soil type (pH, soil moisture and SOC), soil nutrients (total N, total P, C:N, N:P, C:P) and plant litter quantity (litter layer, g m^−2^). Each variable was added as a linear or quadratic term, with and without interactions with individual body mass (*M*). Models were then compared by testing their influence on goodness of fit (Akaike’s Information Criterion, AIC), model likelihood (Chi-square *p* < 0.05) and parsimony (∆AIC > 2 for additional degrees of freedom). Models that met these criteria were tested with the subsequent environmental variables. Pseudo-marginal (fixed effect) and conditional (fixed and random effect) *R*^2^ values for the hierarchical models were calculated using the MuMIn package.

## Results

### Soil animal community composition

**Fig. 1:**
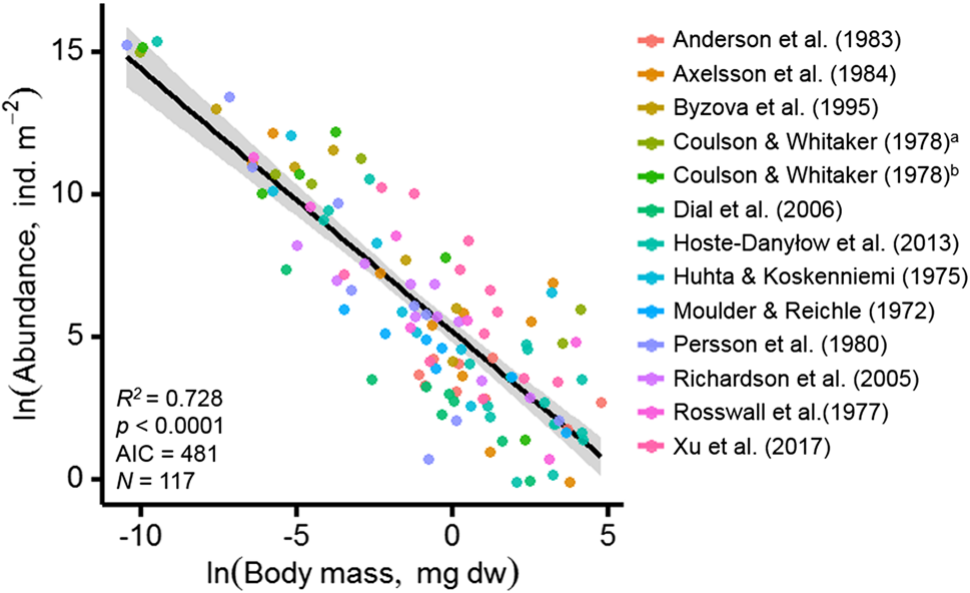
Mass–abundance scaling relationship across thirteen globally distributed soil animal communities (coloured symbols). Abundance (individuals m^−2^) is plotted against body mass (mg dry mass) for the soil animal groups reported (*N* = 117). The best-fitting regression model (black line, with shaded areas showing standard error) was *y* = 5.23 − 0.92*x* (Table S2)

Mass–abundance relationships did not differ across thirteen globally distributed sites (Fig. 1). That is, little support was available for interactions between soil animal body mass and climate (arctic, boreal, temperate or tropical), ecosystem type (forest or grassland) or study site (*N*_study_ = 13) (Table S2).

**Fig. 2:**
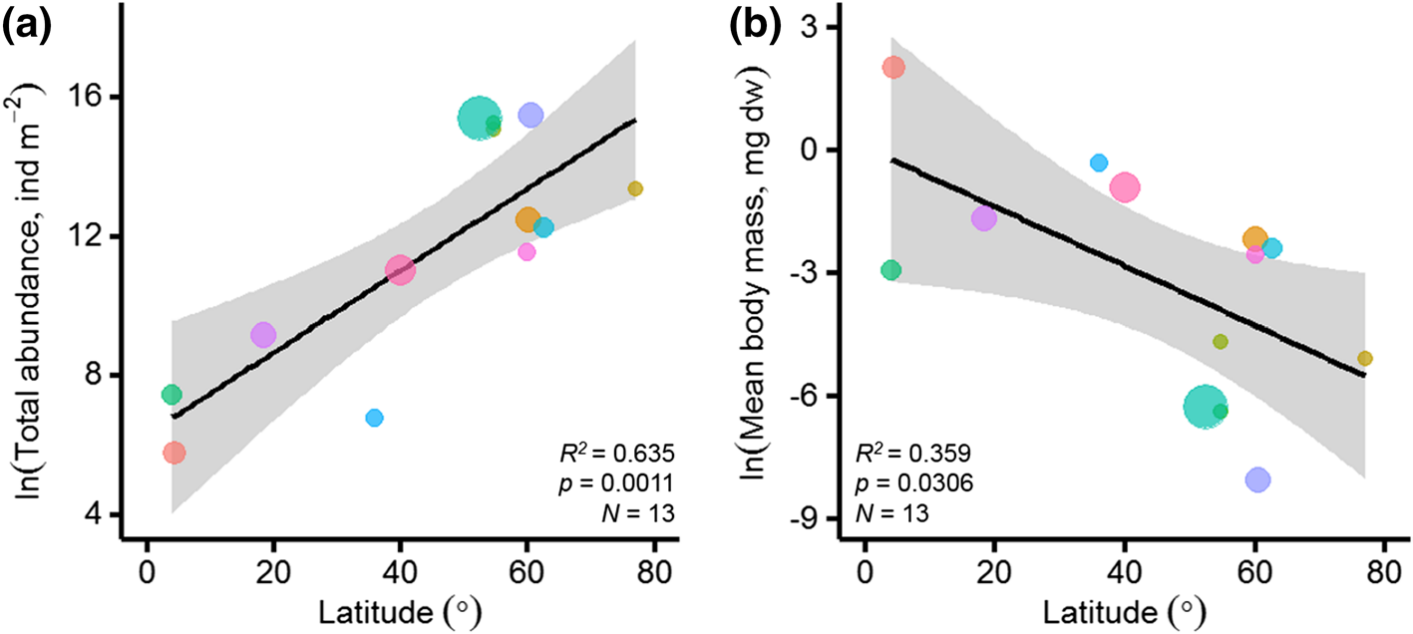
Latitudinal shifts in soil animal community composition, showing **a** the relationship between total soil animal community abundance and **b** mean soil animal body mass across the studies (*N*_study_ = 13) with latitude. The best-fitting regression models (black lines, with shaded areas showing standard error) were **a***y* = 6.34 + 0.12*x*, and **b***y* = 0.07 −v0.07*x*. Symbol colours represent the thirteen studies as in Fig. 1, while symbol diameter represents the number of soil animal groups measured in each study

Soil animal community body-size spectra were, however, affected by latitude (Table S2) suggesting the influence of environmental controls that co-vary with distance from the equator. We illustrate the influence of latitude on total soil animal abundance and mean soil animal body mass in Fig. 2a and b, respectively. Figure 2 reveals latitudinal shifts in the overall body-size spectra of soil animal communities across thirteen globally distributed sites. Specifically, the data reveal that larger soil animal groups (e.g., Coleoptera, Chilopoda, Araneae, Clitellata) become increasingly abundant in low latitude (tropical and some temperate) soils, while high latitude (tundra, boreal and some temperate) soils are dominated by smaller animals (e.g., Nematoda, Collembola, Acari, Enchytraeidae).

### Multiple environmental controls on global soil animal communities

A hierarchical linear mixed effect model was used to test the importance of environmental controls (MAT, MAP, soil moisture, SOC, pH, total N, total P, C:N, N:P, C:P and litter layer) on mass–abundance relationships for each soil animal group (*N*_group_ = 117). Addition of three terms improved the hierarchical model fits in comparison to the null model (Table 3), with the condition that adding an additional term must be met with a goodness of fit of ∆AIC > 2 and Chi-square *p* < 0.05. Mean annual temperature (MAT), soil pH and soil organic carbon (SOC) content met these conditions (Table 3). The final model had an improved goodness of fit to the data and model likelihood compared to the null model (∆AIC = 16.29, Chi-square *p* < 0.0001). The hierarchical model also showed an improved goodness of fit and model likelihood in comparison to the latitude model (∆AIC = 7.98, Chi-square *p* = 0.0026, Table S2). Importantly, the hierarchical model further reveals the interactions between environmental variables underpinning latitudinal shifts in global soil communities.

**Table 3:** Comparison of hierarchical linear mixed effects models used to explain global patterns in soil animal community mass–abundance relationships

Environmental controls		*df*	*R*^2^_m_	*R*^2^_c_	Chi-square *p*	AIC	ΔAIC
Null	ln(*A*) ~ ln(*M*) + (1|Study) + (1|Sample size)	4	0.702	0.799	na	481.02	16.29
Climate	ln(*A*) ~ ln(*M*) × MAT + (1|Study) + (1|Sample size)	6	0.759	0.823	0.003	473.41	8.68
Edaphic	ln(*A*) ~ ln(*M*) × MAT × pH + pH^2^ × SOC + (1|Study) + (1|Sample size)	14	0.805	0.828	0.002	464.73	0

The null model does not include interactions between soil animal body mass (*M*) and environmental variables, while the following models indicate additional terms added to a hierarchical model. Chi-square *p* value < 0.05 indicates increased model likelihood following the addition of each term. Overall goodness of fit is determined by AIC values, where lower AICs indicate a better fit to the data (*N* = 117). ∆AIC’s present the difference in AIC values between the different models and the final hierarchical model (Table S3)

**Fig. 3:**
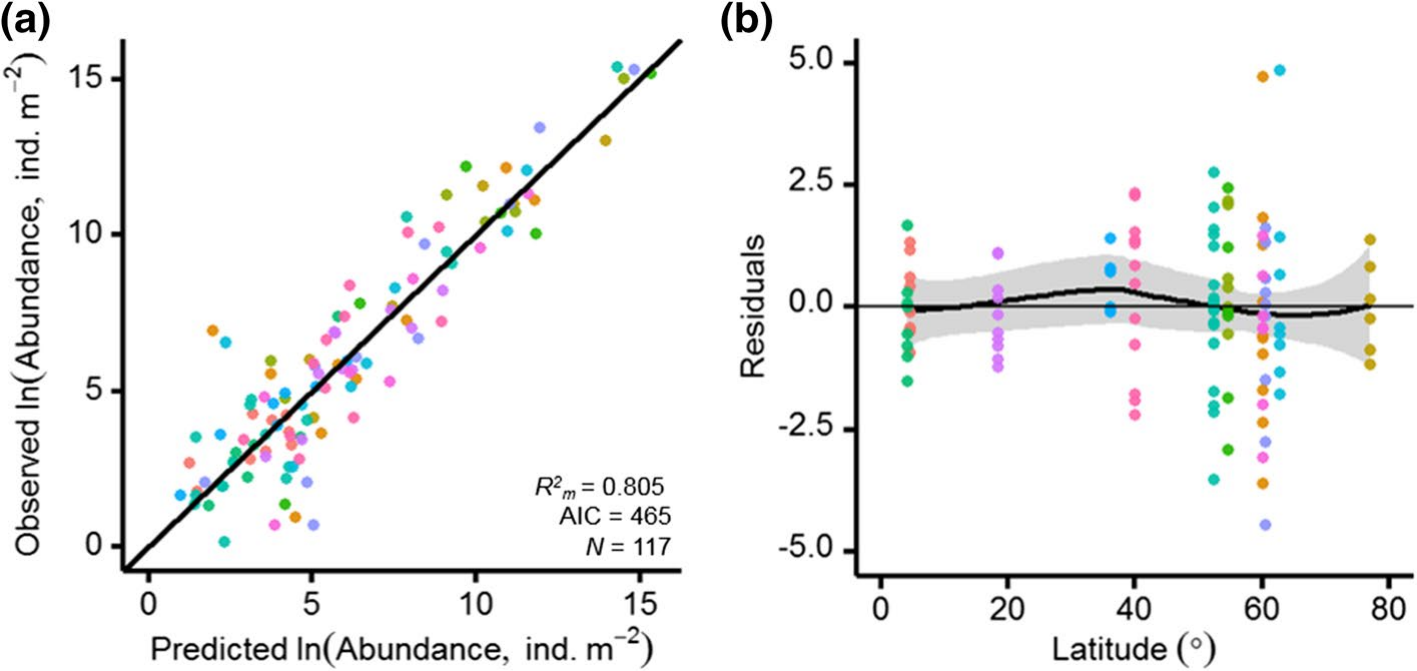
Final hierarchical model (ln(*A*) ~ ln(*M*) × MAT × pH + pH^2^ × SOC + (1|Study) + (1|Sample size)) **a** predictions compared to observed soil animal abundances (*N*_group_ = 117) across thirteen globally distributed sites, and **b** model residuals against latitude, with the lowess fit (black lines, with shaded areas showing standard error) displaying deviations between model predictions and observed abundances

Predicted soil animal group abundances from fixed effects (*M,* MAT, soil pH and SOC) in the final hierarchical model are compared to observed abundances in Fig. 3a. The distribution of the final hierarchical model residuals, against latitude as an independent variable, further demonstrate deviations between model predictions and observed abundances in Fig. 3b. The largest deviations between predicted and observed abundances occurred in high-latitude boreal or arctic soils, and particularly not only for larger soil animal groups (Chilopoda, Diplopoda, Isopoda, Clitellata) but also for Enchytraeidae. Smallest deviations of the final hierarchical model were present for smaller soil animal groups in high-latitude soils, and larger soil animal groups in temperate and tropical soils. The lowess fit in Fig. 3b, however, indicates prediction of average abundances across latitudes, with average soil animal abundance slightly under-predicted (positive residuals) and over-predicted (negative residuals) at mid-latitude and high-latitude sites, respectively.

**Fig. 4:**
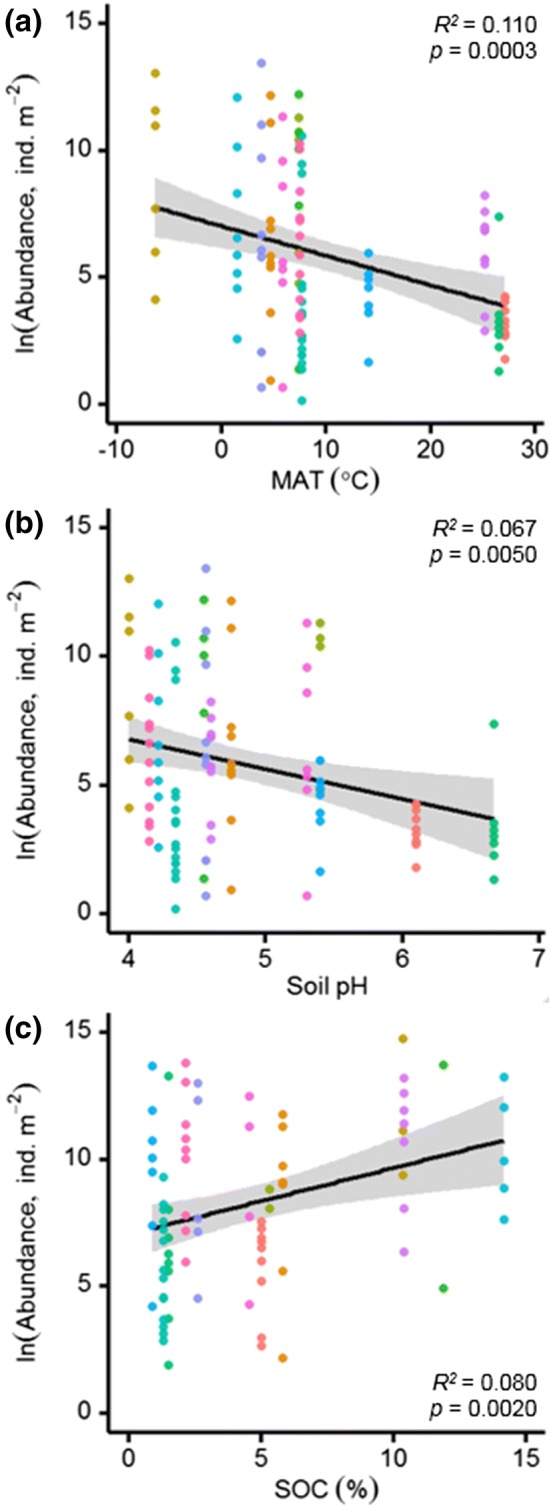
Relationships between soil animal abundances with **a** mean annual temperature (MAT, °C), **b** soil pH and **c** soil organic carbon (SOC, %) content (*N*_group_ = 117, symbol colours represent different studies as in Fig. 1). Best-fitting regression models (black lines, with shaded areas showing standard error) were **a***y* = 14.38 − 0.29*x*; **b***y* = 7.34 − 0.13*x*; **c***y* = 12.16 − 1.28*x* and **d***y* = 4.66 + 0.25*x*

The influence of MAT, soil pH and SOC on soil animal abundances is illustrated in Fig. 4, to provide an assessment of the directionality of relationships between soil animal communities and environmental controls. Results reveal linear declines in abundance with increasing MAT and soil pH (Fig. 4a, b), and an increase in abundance with increasing SOC (Fig. 4c). At low-latitude sites, higher temperatures, less acidic soils with lower SOC contents supported higher abundances of larger soil animals, whereas high-latitude sites with greater abundances of small soil animals were characterised by low temperatures, acidic soils with higher SOC contents. MAT and soil pH show strong correlations with latitude across the thirteen sites, whereas SOC contents were most strongly correlated with total soil N, C:P and N:P ratios (Figure S1).

## Discussion

Our study reveals how the body-size spectra of soil animal communities converge across globally distributed sites (Fig. 1 and Table S2). Latitude showed a stronger relationship with soil animal mass–abundance relationships than climate, ecosystem type, or study, revealing shifts from dominance by small soil animals at high latitudes to greater relative abundances of large soil animals at low latitudes (Fig. 2). We used a hierarchical linear mixed effects model to test the importance of multiple environmental controls in shaping these latitudinal trends in global soil animal communities. Climate and edaphic conditions best explained global variations in soil animal community composition (Table 3), with the largest deviations between predicted and observed abundances occurring in high-latitude soils (Fig. 3). The influence of the fixed effects in the final hierarchical model on soil animal abundances was illustrated to show the directionality and strength of each relationship (Fig. 4). Overall, soil animal abundances declined with increasing MAT and soil pH and increased with SOC contents.

Recent global syntheses of soil communities have identified contrasting environmental controls on the distribution and abundance of soil animal groups of different body size ranges. Nematode abundances, for instance, increase with an increase in SOC content and decline with increasing soil pH at a global scale (Van Den Hoogen et al. 2019). Global earthworm communities, on the other hand, have been more strongly linked to climatic variables (Phillips et al. 2019). Soil acidity also influences global earthworm communities across natural and managed ecosystems, with higher species richness at intermediate soil pH values (Johnston 2019). Relationships between smaller and larger soil animal groups at a global scale broadly follow those identified for soil fungi and bacteria, respectively. Soil bacterial communities, for example, display greater diversity at intermediate pH values (Fierer and Jackson 2006) while fungi often dominate microbial communities in acidic soils (Rousk et al. 2010; Tedersoo et al. 2014). Declines in soil acidity are further expected to cause microbial community composition shifts from fungal to bacterial dominance, leading to greater trophic transfer efficiency to their soil animal consumers (Carrillo et al. 2016). Our study generalises these results to entire soil animal communities, whereby the dominance of small soil animals (e.g., Nematoda, Acari, Collembola) occurs in high-latitude acidic soils with higher SOC contents, and greater abundances of larger soil animals (e.g., Chilopoda, Coleoptera, Clitellata) occur at mid- to low latitudes in more neutral soils with lower SOC contents.

The influence of climate, and specifically MAT, on soil animal community composition can be understood through the temperature and size dependence of multiple biological processes (Dell et al. 2011). Soil animals are known to display varying temperature sensitivities, with smaller soil animals displaying a greater increase in metabolic rate with increasing temperature (Johnston and Sibly 2018). Smaller animals thus require a greater relative increase in energy from food resources to meet higher metabolic demands with increasing temperature, and so we would expect their populations to become less abundant in warmer climates. In this study we find that smaller soil animal abundances declined in warmer low-latitude climates, while larger soil animals were absent, or present at low abundances, in colder high-latitude climates (Fig. 2). These latitudinal shifts in the body-size spectra of soil animal communities suggest the importance of both soil animal temperature sensitivities and resource availability in shaping their global distributions and abundances. Indeed, soil warming experiments have demonstrated direct impacts of increasing temperature on soil animal communities within biomes (Briones et al. 2009; Bokhorst et al. 2012), but highly variable responses are thought to be driven by the availability of different food resources in mid- and high-latitude soils (Aerts 2006; Robinson et al. 2018).

We hypothesised that soil nutrients would best describe soil animal body-size spectra, due to the strong relationships between nutrient limitation and plant production above- and belowground. Nutrient availability or stoichiometry was not identified as fixed effects in the hierarchical model. However, soil acidity (or pH) strongly influences the availability of multiple soil nutrients (Binkley and Vitousek 1989). Across the sites analysed here, for instance, soil pH was correlated with SOC, total N and P, soil C:N and C:P ratios while SOC was correlated with soil pH, soil C:P and N:P (Figure S1). Soil acidity has been identified as a key factor influencing soil animal communities at large scales (Mulder and Elser 2009; Johnston 2019), with soil macrofauna (e.g., Oligochaeta) largely restricted to soils with pH values above 3.5 (Schlaghamerský et al. 2014). A lack of clear relationships between soil nutrients (N and P) and soil animal communities, both here and in other studies, could indicate the overlooked importance of additional soil nutrients which correlate with soil pH. In temperate forests, for instance, litter calcium concentrations among 14 tree species resulted in large changes in soil acidity and earthworm abundance and diversity, 30 years after establishment (Reich et al. 2005). Soil pH, therefore, likely reflects the availability of multiple nutrients, the optimal ranges for which will differ across soil animal groups.

Evidence supporting the influence of temperature, soil pH and SOC on soil animal communities is available from global decomposition studies. Globally, climate and litter quality (C:N ratios, which correlate with soil pH and SOC) are primary drivers of litter decomposition rates (Parton et al. 2007), while soil animals display variable positive effects on decomposition rates across biomes (Wall et al. 2008). Soil animals have a greater positive effect on litter decomposition rates in warm and wet, compared to cold and dry, ecosystems (García‐Palacios et al. 2013). These observations have been explained in terms of low temperature and moisture constraints on biological activity. Our study suggests that temperature, soil pH and SOC controls on soil animal body-size spectra may play an important role in global patterns of litter decomposition rates because larger soil animals become more abundant where the effect of soil animals on decomposition rates is greatest (e.g., temperate and tropical soils). Greater litter decomposition rates then feedback to plant communities by enhancing nutrient availability for plant uptake (Swift et al. 1998). On the other hand, smaller soil animals show preference for acidic nutrient-limited soils, while reduced litter decomposition by small soil animals at low temperatures leads to soil organic matter accumulation and slow nutrient turnover rates which restrain plant productivity (Petersen and Luxton 1982; Loranger et al. 2001).

Unravelling the ecological mechanisms that underpin soil communities requires comprehensive datasets at multiple levels of biological organisation and ecological scales. The data compiled in this study were purposefully limited to include only studies that report entire soil animal communities, rather than compiling soil animal group data from various locations and sources as in previous syntheses (Petersen and Luxton 1982; Fierer et al. 2009). However, limitations of the dataset constrain robust analysis of global patterns. For instance, there is a general deficit of soil animal studies in mediterranean, subtropical and tropical regions, particularly in comparison to temperate ecosystems. Smaller soil animal groups also tend to be less well studied in warmer climates. Cross-biome studies of seasonal soil microbial and animal community dynamics together with plant and environmental variables are needed to fill these key knowledge gaps. Although such studies are time consuming, new information is crucial for improving our quantitative understanding on the environmental controls shaping soil communities at the globe scale.

In summary, our study reveals the importance of temperature, soil pH and SOC as key drivers of soil animal body-size spectra at a global scale. We suggest that temperature influences soil community composition through temperature- and size-dependent metabolic demands, and soil pH and SOC reflect the availability of multiple nutrients that drive resource availability for different soil animal groups. Greater resolution global datasets of soil animal communities are needed to uncover the mechanisms underpinning environmental controls on soil communities. Such knowledge is needed to develop predictive soil ecology models capable of forecasting the effects of environmental changes on soil communities and ecosystem functions.

## Electronic supplementary material

Below is the link to the electronic supplementary material.

Supplementary file1 (DOCX 75 kb)

## Data Availability

The dataset compiled and analysed in this study is available from Dryad (10.5061/dryad.6djh9w0xs).
