# Supplementary material for: Multiple environmental controls explain global patterns in soil animal communities
Source: Oecologia. 2020 Apr 7;192(4):1047–56. doi: 10.1007/s00442-020-04640-w (PMC7165188; doi:10.1007/s00442-020-04640-w)
Supplement: Supplementary file 1 — Supplementary file1 (DOCX 75 kb) [file 442_2020_4640_MOESM1_ESM.docx]

**SUPPLEMENTARY INFORMATION**

| **Table S1.** Additional sources of derived environmental variables for each soil community study. | | | |
| --- | --- | --- | --- |
| **Study** | **Variable** | **Value** | **Source** |
| Axelsson, Lohm and Persson (1984) | Litter layer | 358.4 g m^-2^ | Hytteborn (1975)  Fig. 30 & Table 25a |
|  | SOC | 5.83 % (10.04 × 0.58) |  |
|  | Soil N | 3.2 % |  |
|  | Soil P | 117 mg kg^-1^ | Lundell (1987) Figure 2 |
| Byzova, Uvarov and Petrova (1995) | Litter layer | 220 g m^-2^ | Klekowski and Opaliński (1986) Table 1 |
|  | SOC  Soil C:N  pH | 10.36 %  13.05  4 | Dziadowiec, Gonet and Plichta (1994)  Table 1 |
|  | Soil P | 1460 mg kg^-1^ | Godzik (1991) Table 2 |
| Coulson and Whittaker (1978) Acid | Litter layer | 217.8 g m^-2^ | Welch and Rawes (1964) Table 4a |
|  | Soil P | 0.16 % | Latter, Cragg and Heal (1967) Table 2 |
| Coulson and Whittaker (1978) Limestone | Litter layer | 668.5 g m^-2^ | Forrest and Smith (1975)  Tables 7 & 8 |
|  | Soil P | 0.14 % | Latter, Cragg and Heal (1967) Table 2 |
| Dial *et al.* (2006) | pH | 6.67 | Berry *et al.* (2008) Table 2 |
|  | Litter layer  SOC | 4.8 Mg C ha^-1^ (960 g m^-2^)  1.49 % | Saner *et al.* (2012) Figure 5  Table 2 |
|  | Soil N  Soil P | 0.23 %  177.8 mg kg^-1^ | Nussbaum, Anderson and Spencer (1995)  Table 2 |
| Hoste-Danyłow *et al.* (2013) | Litter layer | 147 g m^-2^ | Topp *et al.* (2006) |
|  | SOC  Soil N  Soil P  pH | 1.32 %  0.06 %  90.5 mg kg^-1^  4.34 | Kurek, Kapusta and Holeksa (2014)  Figure 2 – Reference Area |
| Huhta and Koskenniemi (1975) | Litter layer | 375 g m^-2^ | Huhta (1976) pp. 76-77 |
|  | SOC  Soil P  pH | 14.17 %  0.03 %  4.22 | Piirainen *et al.* (2004) Table 2 |
|  | Soil N | 0.41 % | Piirainen *et al.* (2002) Table 2 |
| Moulder and Reichle (1972) | Litter layer  Soil N  Soil P  pH | 22860 kg C ha^-1^ (1143 g m^-2^)  3500 mg kg^-1^  960 mg kg^-1^  5.4 | Johnson and Todd (1987)  Tables 1 & 2 |
|  |  |  |  |
| Rosswall, Persson and Lohm (1977) | Soil C:N | 8.3 | Paustian, Parton and Persson (1992) Table 4 |
|  | Soil N  pH | 0.45 %  5.3 | Hansson and Fogelfors (2000) Table 1 |
|  | Soil P | 186 mg kg^-1^ | Sundström, Magnusson and Hånell (2000) Table 3 |
| Persson *et al.* (1980) | Soil P | 117 mg kg^-1^ | Lundell (1987) Figure 2 |
| Richardson, Richardson and Soto-Adames (2005) | SOC  Soil N  pH | 10.4 %  0.65 %  4.6 | Erickson, Keller and Davidson (2001) Table 2 |
|  | Soil P | 149 mg kg^-1^ | Frizano *et al.* (2002) Table 1 |
| Xu *et al.* (2017) | SOC  Soil N  Soil P  pH | 2.15 % (3.70 × 0.58)  930 mg kg^-1^  380 mg kg^-1^  4.15 | Liu *et al.* (2012) Table 2 |

| **Table S2.** ANOVA results for linear regression analyses of ln(abundance (*A,* number m^-2^)) on ln(body mass (*M*, mg dry weight)) for the soil animal groups across thirteen globally distributed sites. | | | | | |
| --- | --- | --- | --- | --- | --- |
| **ANOVA** | **Degrees of freedom** | **Sum of squares** | **Mean square** | ***F*** | ***p*** |
| **Null model: AIC = 492.82** | |  |  |  |  |
| ln(Mass, mg) | 1 | 1175.93 | 1175.93 | 307.84 | < 0.0001 |
| Residuals | 115 | 439.30 | 3.82 |  |  |
| **Ecosystem type: AIC = 485.54** | |  |  |  |  |
| ln(Mass, mg) | 1 | 1175.93 | 1175.93 | 333.10 | < 0.0001 |
| Ecosystem type | 1 | 39.90 | 39.90 | 11.30 | 0.0011 |
| *M* × Eco. Type | 1 | 0.48 | 0.48 | 0.13 | 0.7138 (NS) |
| Residuals | 113 | 398.92 | 3.53 |  |  |
| **Climate: AIC = 483.48** | |  |  |  |  |
| ln(Mass, mg) | 1 | 1175.93 | 1175.93 | 350.18 | < 0.0001 |
| Climate | 3 | 64.20 | 21.40 | 6.37 | 0.0005 |
| *M* × Climate | 3 | 9.07 | 3.02 | 0.90 | 0.4436 (NS) |
| Residuals | 109 | 366.03 | 3.36 |  |  |
| **Study: AIC = 472.16** | |  |  |  |  |
| ln(Mass, mg) | 1 | 1175.93 | 1175.93 | 438.06 | < 0.0001 |
| Study | 12 | 151.07 | 12.59 | 4.68 | < 0.0001 |
| *M* × Study | 12 | 43.94 | 3.66 | 0.20 | 0.1978 (NS) |
| Residuals | 91 | 244.28 | 2.68 |  |  |
| **Latitude: AIC = 471.85** | |  |  |  |  |
| ln(Mass, mg) | 1 | 1175.93 | 1175.93 | 377.52 | < 0.0001 |
| Latitude | 1 | 60.84 | 60.84 | 19.53 | < 0.0001 |
| Latitude^2^ | 1 | 10.86 | 10.86 | 3.49 | 0.0645 (NS) |
| *M* × Latitude | 1 | 18.73 | 18.73 | 6.01 | 0.0157 |
| Residuals | 112 | 348.86 | 3.11 |  |  |

| **Table S3.** Fixed and random effects, and ANOVA for the hierarchical linear mixed effects model: ln(*A*) ~ ln(*M*) × MAT × pH + pH^2^ × SOC + (1\|Study) + (1\|N), where *A* is average soil animal group abundance (individuals m^-2^), *M* is average soil animal group body mass (mg dw), MAT is mean annual temperature (°C) and SOC is soil organic carbon (%). The model was fitted to the mass-abundance dataset for thirteen globally distributed soil animal communities (N = 117). Results presented are for Type III ANOVA, which accounts for the effects of environmental variables after other environmental variables and their interactions. | | | | | |
| --- | --- | --- | --- | --- | --- |
| **Fixed effects** | **Estimate** | **SE** | **Random effects** | **Variance** | **SD** |
| Intercept | 72.71 | 19.19 | Study | 0.339 | 0.583 |
| ln(*M*) | -1.911 | 0.652 | Sample size (N) | 0.000 | 0.000 |
| MAT (°C) | 1.041 | 0.299 | Residual | 2.506 | 1.582 |
| Soil pH | -28.28 | 8.120 |  |  |  |
| Soil pH^2^ | 2.938 | 0.855 |  |  |  |
| SOC (%) | -0.888 | 0.276 |  |  |  |
| ln(*M*) × MAT | 0.028 | 0.039 |  |  |  |
| ln(*M*) × Soil pH | 0.204 | 0.143 |  |  |  |
| MAT × Soil pH | -0.236 | 0.063 |  |  |  |
| Soil pH^2^ × SOC | 0.050 | 0.014 |  |  |  |
| ln(*M*) × MAT × Soil pH | -0.004 | 0.008 |  |  |  |
| **ANOVA** | **Degrees of freedom** | **Sum of squares** | **Mean square** | ***F*** | ***p*** |
| ln(*M*) | 1 | 21.48 | 21.48 | 8.583 | 0.0042 |
| MAT (°C) | 1 | 30.40 | 30.40 | 12.15 | 0.0008 |
| Soil pH | 1 | 30.35 | 30.35 | 12.13 | 0.0009 |
| Soil pH^2^ | 1 | 29.52 | 29.52 | 11.80 | 0.0010 |
| SOC (%) | 1 | 25.95 | 25.95 | 10.37 | 0.0023 |
| ln(*M*) × MAT | 1 | 1.272 | 1.272 | 0.508 | 0.4774 |
| ln(*M*) × Soil pH | 1 | 5.111 | 5.111 | 2.042 | 0.1559 |
| MAT × Soil pH | 1 | 34.63 | 34.63 | 13.84 | 0.0004 |
| Soil pH^2^ × SOC | 1 | 31.58 | 31.58 | 12.62 | 0.0009 |
| ln(*M*) × MAT × Soil pH | 1 | 0.640 | 0.640 | 0.256 | 0.6142 |


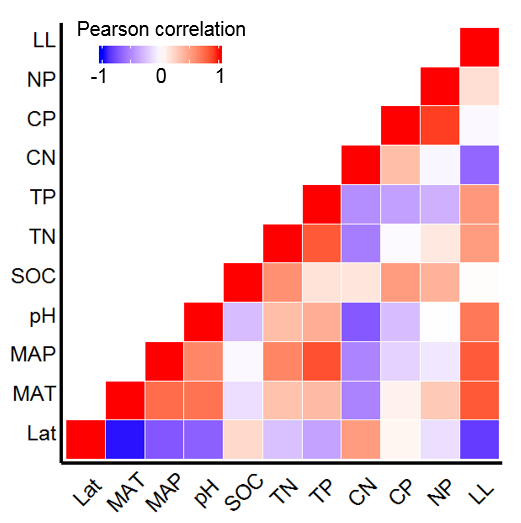


**Figure S1.** Heatmap of correlations between environmental variables compiled from thirteen globally-distributed sites. Lat is latitude (° North), MAT is mean annual temperature (° C), MAP is mean annual precipitation (mm), SOC is soil organic carbon (%), TN is total nitrogen (%), TP is total phosphorus (%), CN, CP and NP are ratios between soil carbon (C), nitrogen (N) and phosphorus (P) and LL is litter layer (g dw m^-2^).

**References**

Axelsson, B., Lohm, U. & Persson, T. (1984) Enchytraeids, lumbricids and soil arthropods in a northern deciduous woodland–A quantitative study. *Ecography,* **7,** 91-103.

Berry, N.J., Phillips, O.L., Ong, R.C. & Hamer, K.C. (2008) Impacts of selective logging on tree diversity across a rainforest landscape: the importance of spatial scale. *Landscape Ecology,* **23,** 915-929.

Byzova, J.B., Uvarov, A.V. & Petrova, A.D. (1995) Seasonal changes in communities of soil invertebrates in tundra ecosystems of Hornsund, Spitsbergen. *Polish Polar Research,* **16,** 245-266.

Coulson, J. & Whittaker, J. (1978) Ecology of moorland animals. *Production ecology of British moors and montane grasslands*, pp. 52-93. Springer.

Dial, R.J., Ellwood, M.D.F., Turner, E.C. & Foster, W.A. (2006) Arthropod Abundance, Canopy Structure, and Microclimate in a Bornean Lowland Tropical Rain Forest. *Biotropica,* **38,** 643-652.

Dziadowiec, H., Gonet, S. & Plichta, W. (1994) Properties of humic acids of Arctic tundra soils in Spitsbergen. *Polish Polar Research,* **15,** 71-81.

Erickson, H., Keller, M. & Davidson, E.A. (2001) Nitrogen oxide fluxes and nitrogen cycling during postagricultural succession and forest fertilization in the humid tropics. *Ecosystems,* **4,** 67-84.

Forrest, G. & Smith, R. (1975) The productivity of a range of blanket bog vegetation types in the northern Pennines. *The Journal of Ecology,* **63,** 173-202.

Frizano, J., Johnson, A.H., Vann, D.R. & Scatena, F.N. (2002) Soil Phosphorus Fractionation during Forest Development on Landslide Scars in the Luquillo Mountains, Puerto Rico 1. *Biotropica,* **34,** 17-26.

Godzik, B. (1991) Heavy metals and macroelements in the tundra of southern Spitsbergen: the effect of little auk Alle alle (L.) colonies. *Polar Research,* **9,** 121-131.

Hansson, M. & Fogelfors, H. (2000) Management of a semi‐natural grassland; results from a 15‐year‐old experiment in southern Sweden. *Journal of vegetation science,* **11,** 31-38.

Hoste-Danyłow, A., Ilieva-Makulec, K., Olejniczak, I., Hajdamowicz, I., Stańska, M., Marczak, D., Wytwer, J., Faleńczyk-Koziróg, K. & Ulrich, W. (2013) The shape of the intraspecific metabolic-rate–body-size relationship affects interspecific biomass and abundance distributions of soil animals within a forest ecosystem. *Annales Zoologici Fennici,* **50,** 289-302.

Huhta, V. (1976) Effects of clear-cutting on numbers, biomass and community respiration of soil invertebrates. *Annales Zoologici Fennici*, pp. 63-80. JSTOR.

Huhta, V. & Koskenniemi, A. (1975) Numbers, biomass and community respiration of soil invertebrates in spruce forests at two latitudes in Finland. *Annales Zoologici Fennici,* **12,** 164-182.

Hytteborn, H. (1975) Deciduous woodland at Andersby, eastern Sweden: above-ground tree and shrub production. Doctoral dissertation, Sv. växtgeografiska sällsk.

Johnson, D. & Todd, D. (1987) Nutrient export by leaching and whole-tree harvesting in a loblolly pine and mixed oak forest. *Plant and Soil,* **102,** 99-109.

Klekowski, R.Z. & Opaliński, K.W. (1986) Matter and energy flow in Spitsbergen ornithogenic tundra. *Polar Research,* **4,** 187-197.

Kurek, P., Kapusta, P. & Holeksa, J. (2014) Burrowing by badgers (Meles meles) and foxes (Vulpes vulpes) changes soil conditions and vegetation in a European temperate forest. *Ecological Research,* **29,** 1-11.

Latter, P.M., Cragg, J. & Heal, O. (1967) Comparative studies on the microbiology of four moorland soils in the northern Pennines. *The Journal of Ecology,* **55,** 445-464.

Liu, L., Gundersen, P., Zhang, T. & Mo, J. (2012) Effects of phosphorus addition on soil microbial biomass and community composition in three forest types in tropical China. *Soil Biology and Biochemistry,* **44,** 31-38.

Lundell, Y. (1987) Nutrient variation in forest soil samples due to time of sampling and method of storage. *Plant and Soil,* **98,** 363-375.

Moulder, B.C. & Reichle, D.E. (1972) Significance of Spider Predation in the Energy Dynamics of Forest-Floor Arthropod Communities. *Ecological Monographs,* **42,** 473-498.

Nussbaum, R., Anderson, J. & Spencer, T. (1995) Factors limiting the growth of indigenous tree seedlings planted on degraded rainforest soils in Sabah, Malaysia. *Forest Ecology and Management,* **74,** 149-159.

Paustian, K., Parton, W.J. & Persson, J. (1992) Modeling soil organic matter in organic-amended and nitrogen-fertilized long-term plots. *Soil science society of America journal,* **56,** 476-488.

Persson, T., Bååth, E., Clarholm, M., Lundkvist, H., Söderström, B.E. & Sohlenius, B. (1980) Trophic Structure, Biomass Dynamics and Carbon Metabolism of Soil Organisms in a Scots Pine Forest. *Ecological Bulletins,* **32,** 419-459.

Piirainen, S., Finér, L., Mannerkoski, H. & Starr, M. (2002) Effects of forest clear-cutting on the carbon and nitrogen fluxes through podzolic soil horizons. *Plant and Soil,* **239,** 301-311.

Piirainen, S., Finér, L., Mannerkoski, H. & Starr, M. (2004) Effects of forest clear-cutting on the sulphur, phosphorus and base cations fluxes through podzolic soil horizons. *Biogeochemistry,* **69,** 405-424.

Richardson, B.A., Richardson, M.J. & Soto-Adames, F.N. (2005) Separating the effects of forest type and elevation on the diversity of litter invertebrate communities in a humid tropical forest in Puerto Rico. *Journal of Animal Ecology,* **74,** 926-936.

Rosswall, T., Persson, T. & Lohm, U. (1977) Energetical significance of the annelids and arthropods in a Swedish grassland soil. *Ecological Bulletins,* **23,** 1-211.

Saner, P., Loh, Y.Y., Ong, R.C. & Hector, A. (2012) Carbon stocks and fluxes in tropical lowland dipterocarp rainforests in Sabah, Malaysian Borneo. *PLoS ONE,* **7,** e29642.

Sundström, E., Magnusson, T. & Hånell, B. (2000) Nutrient conditions in drained peatlands along a north-south climatic gradient in Sweden. *Forest Ecology and Management,* **126,** 149-161.

Topp, W., Kappes, H., Kulfan, J. & Zach, P. (2006) Litter-dwelling beetles in primeval forests of Central Europe: does deadwood matter? *Journal of Insect Conservation,* **10,** 229-239.

Welch, D. & Rawes, M. (1964) The early effects of excluding sheep from high-level grasslands in the North Pennines. *Journal of Applied Ecology,* **1,** 281-300.

Xu, G., Lin, Y., Zhang, S., Zhang, Y., Li, G. & Ma, K. (2017) Shifting mechanisms of elevational diversity and biomass patterns in soil invertebrates at treeline. *Soil Biology and Biochemistry,* **113,** 80-88.
